# Clinical Characterization of Anti-GQ1b Antibody Syndrome in Childhood

**DOI:** 10.3389/fped.2021.649053

**Published:** 2021-04-29

**Authors:** Lianhong Cai, Zhanqi Hu, Jianxiang Liao, Siqi Hong, Lingyu Kong, Li Chen, Yetao Luo, Tingsong Li, Li Jiang

**Affiliations:** ^1^Department of Neurology, Children's Hospital of Chongqing Medical University, Ministry of Education Key Laboratory of Child Development and Disorders, National Clinical Research Center for Child Health and Disorders (Chongqing), International Science and Technology Cooperation Base of Child Development and Critical Disorders, Chongqing Key Laboratory of Pediatrics, Chongqing, China; ^2^Department of Neurology, Shenzhen Children's Hospital, Shenzhen, China; ^3^Department of and Clinical Epidemiology and Biostatistics, Children's Hospital of Chongqing Medical University, Chongqing, China

**Keywords:** Miller Fisher syndrome, pediatric, anti-GQ1b antibody, Guillain-Barré syndrome, diangnosis, immunotherapy

## Abstract

**Objective:** To delineate the comprehensive clinical features of anti-GQ1b antibody syndrome in childhood.

**Methods:** The clinical data of children diagnosed with anti-GQ1b antibody syndrome at two Chinese tertiary pediatric neurology centers were collected and analyzed. We also conducted a systematic literature review on anti-GQ1b antibody syndrome in children.

**Results:** This study included 78 children with anti-GQ1b antibody syndrome, consisting of 12 previously unreported cases from the two Chinese centers. The median onset age was 10 years (range, 2–18 years). The most common phenotype was acute ophthalmoparesis (32%), followed by classic Miller Fisher syndrome (15%), and Bickerstaff brainstem encephalitis (12%). External ophthalmoplegia (48%), sensory disturbance (9%), and bulbar palsy (9%) were the three most frequent onset symptom manifestations. Brain or spinal lesions on MRI and abnormal recordings by nerve conduction study were present in 18% (12/68) and 60% (27/45) of cases, respectively. There was CSF albuminocytologic dissociation in 34% of the patients (23/68). IV immunoglobulin alone or combined with steroids or plasma exchange was administered to 58% of patients (42/72). We did not find a significant correlation between early improvement up to 3 months and age onset and phenotype. All patients showed different degrees of recovery, and 81% (57/70) had complete recovery within 1 year.

**Conclusions:** Acute ophthalmoparesis and classic Miller Fisher syndrome are the most common phenotypes of anti-GQ1b antibody syndrome in childhood. The majority of patients show good response to immunotherapy and have favorable prognosis.

## Introduction

Since the discovery of anti-GQ1b antibody in typical Miller Fisher syndrome (MFS) in 1992 (1), Bickerstaff brainstem encephalitis (BBE), acute ophthalmoplegia (AO), and other variants of MFS and Guillain-Barré syndrome (GBS) have been associated with anti-GQ1b antibody (2). As MFS, GBS, BBE, and AO are closely related and form a continuous range, as well as the serum-positive anti-GQ1b antibody status among the aforementioned syndromes, a more inclusive nomenclature, i.e., “anti-GQ1b antibody syndrome” has been proposed to include the common serological profile when referring to the clinical syndromes described by both Bickerstaff and Fisher (2, 3).

Chiba et al. first discovered immunoglobulin G (IgG) anti-GQ1b antibodies in typical MFS (1). Subsequently, the phenotypes of anti-GQ1b antibody syndrome have been expanded to acute post-infectious ophthalmoplegia without ataxia (atypical MFS) (4), ataxic GBS (5), pharyngeal–cervical–brachial weakness (PCBW) (6), and other subtypes (7). As for the pediatric case, although Kikuchi et al. first reported a child presented as atypical MFS without ataxia in 1997 (8), the clinical characteristics of pediatric anti-GQ1b antibody syndrome is still to be elucitaede due to the rarity of this syndrome. Additionally, GBS and MFS were classified as parallel syndromes based on current understanding of the common pathophysiological profiles of each disease in 2014 (9). Therefore, a thorough re-evaluation of the subtypes of anti-GQ1b antibody syndrome in childhood under the new framework is essential.

In the present study, we analyze the clinical characteristics and evolution of 12 cases from two tertiary pediatric neurology centers in China, and summarize all reports of anti-GQ1b antibody syndrome in children published to date, with the aim of providing a comprehensive picture of pediatric anti-GQ1b antibody syndrome.

## Methods

### Standard Protocol Approvals, and Patient Consents

Written informed consent for participation in the study was obtained from the patients' legal guardians. The Institutional Review Boards of Children's Hospital of Chongqing Medical of University and Shenzhen Children's Hospital approved the study.

### Previously Unreported Patients

Twelve previously unreported patients diagnosed with anti-GQ1b antibody syndrome were from China: seven were from the Children's Hospital of Chongqing Medical University, and five were from Shenzhen Children's Hospital. The children were identified from January 2017 up to June 2020. All diagnoses met the criteria of anti-GQ1b antibody syndrome proposed by Wakerley et al.: a continuous spectrum disease includs MFS, GBS, BBE, and acute ophthalmoparesis et al. characterized by positive anti-GQ1b IgG in serum (2, 3, 9). During this period, 467 patients in total were diagnosed with GBS, and underwent blot analysis for the presence of anti-ganglioside antibody in serum with cerebral spinal fluid (CSF) or not. We collected all clinical data including neurological presentation, electrophysiology, MRI findings, CSF analysis, treatment, and prognosis.

### Systematic Review

The other cases with anti-GQ1b antibody syndrome were identified from published data. We performed a systematic search of MEDLINE, Embase, and Web of Science from 1992 to March 2020 using “GQ1b” AND “child” on June 28, 2020. Articles not available in English or Chinese were excluded. We extracted and summarized the aforementioned clinical data in the included reports. The individual diagnoses were reassessed using the new diagnosis classification by Wakerley et al. (Table 1) (9). All the included articles were independently reviewed by the authors (LC, ZH, and TL).

**Table 1:** Diagnostic criteria of anti-GQ1b antibody syndrome in this study.

**Classification**	**Core clinical features**	**Incomplete form**	**Overlap syndrome**
GBS	Weakness and areflexia/hyporeflexia in all four limbs		AO/GBS: overlap with ophthalmoplegia
PCBW	Oropharyngeal, neck and arm weakness and arm areflexia/hyporeflexia; leg weakness (–)	APW: arm and neck weakness (–)	AO/PCBW: overlap with ophthalmoplegia MFS/PCBW: overlap with ataxia, ophthalmoplegia
PGBS	Leg weakness and leg areflexia/hyporeflexia; arm weakness (–)		
BWP	Facial weakness and limb areflexia/hyporeflexia; Ophthalmoplegia (–); ataxia (–); limb weakness (–)		AO/BWP: overlap with ophthalmoplegia
MFS	Ophthalmoplegia, ataxia, and areflexia/hyporeflexia; limb weakness (–); hypersomnolence (–)	AO: ataxia (–) AAN: ophthalmoparesis (–) AP: ptosis	MFS/GBS: overlap with limb weakness MFS/BWP: overlap with facial weakness
BBE	Hypersomnolence and ophthalmoplegia and ataxia; limb weakness (–)	AAH: ophthalmoparesis (–)	BBE/GBS: overlap with limb weakness BBE/BWP: overlap with facial weakness

*AAH, acute ataxic hypersomnolence; AAN, acute ataxic neuropathy; AO, acute ophthalmoparesis; AP, acute ptosis; APW, acute pharyngeal weakness; BBE, bickerstaff brainstem encephalitis; BWP, bifacial weakness with paraesthesias; GBS, Guillain–Barré syndrome; MFS, Miller Fisher syndrome; PCBW, pharyngeal–cervical–brachial weakness; PGBS, paraparetic GBS*.

### Statistical Analyses

To assess the factors affecting prognosis, multifactorial binomial logistic regression was performed for two groups: complete recovery within 3 months and incomplete recovery within 3 months. Long-term prognosis and associated factors can not be analyzed statistically because of the incomplete data from different publications at variant timepoins. The candidate predictors were rank or categorical variables, including sex, age, history of antecedent infection, CSF albuminocytologic dissociation (ACD), nerve conduction study (NCS), cranial and spinal MRI, diagnostic subtype, and treatment. Chi-square test and the fisher's exact test were used in variable selection and in the comparison of clinical features in two groups, while Mann–Whitney's U-test was used in the circumstance that needed a non-parametric test. We excluded the measured variables with coefficient *p*-values of >0.2 in univariate screening. IBM SPSS 24 statistical software (IBM, Armonk, NY) was used for all statistical analyses.

## Results

### Clinical Data of 12 Unpublished Chinese Patients

The detailed demographic and clinical profile were described in Supplementary Table 1. The onset age of this cohort ranged from 2 years 6 months to 13 years of age (median age: 6 years 4.5 months). The ratio of male:female was 8:4. Seven out of twelve cases had antecedent infection as upper respiratory tract infection. The most prevalent onset symptom was external ophthalmoplegia (30%, 6/20), followed by limb weakness (25%, 5/20) and sensory disturbaces (20%, 4/20). Regarding to the phenotype, there were 3 cases with sole GBS and MFS, repesctively, as well as 1 case with BBE. The other 5 subjects were diagnosed as overlap syndrome. All the patients were administrated with IVIG combining with corticosteroids or not and achieved full recovery at from 15 to 210 days after the onset. Table 2 demonstrated the comparision of clinical features of cases from our two tertiatery centers and the reported cases. In order to dinineate the comprehensive picture of pediatric anti-GQ1b antibody syndrome, the clinical data of all the 12 individuals were combined with the published study to be analyzed systematically.

**Table 2:** Clinical features of unpublished cases and previously reported childhood cases.

	**Published data**	**Present study**
	**(*n =* 66)**	**(*n =* 12)**
Age of onset (yr)	9.4 ± 4.5	6.6 ± 3.5
Male:Female	42:15^a^	8:4
Preceding infection	47/66 (71.2%)	7/12 (58.3%)
Onset symptom		
External ophthalmoplegia	43/82 (52.4%)	6/20 (30.0%)
Sensory disturbaces	6/82 (7.3%)	4/20 (20.0%)
Bulbar palsy	6/82 (7.3%)	3/20 (15.0%)
Limb weakness	3/82 (3.7%)	5/20 (25.0%)
Top three phenotypes		
	AO (25/66, 37.9%)	MFS (3/12, 25.0%)
	MFS (9/66, 13.6%)	BBE/GBS (2/12, 16.7%)
	BBE (8/66, 12.1%)	PGBS (2/12, 16.7%)
Antiganglioside antibody positivity rather than GQ1b	14/66 (21.2%)	8/12 (66.7%)
ACD in CSF study	16/56^a^ (28.6%)	7/12 (58.3%)
NCS abnormality	16/33^a^ (48.5%)	11/12 (91.7%)
MRI abnormality	11/56^a^ (19.6%)	1/12 (8.3%)
Treatment		
IVIG only	21/66 (31.8%)	5/12 (41.6%)
IVIG+ steroids	4/66 (6.0%)	7/12 (58.3%)
Supportive care only	21/66 (31.8%)	0/12 (0%)
Other treatments	20/66 (30.3%)	0/12 (0%)
CR within 3 months	19/66 (28.8%)	8/12 (66.7%)

a *statistics using the number of patients who were available for each test; ACD, Albuminocytologic dissociation; AO, acute ophthalmoplegia; BBE, Bickerstaff brainstem encephalitis; CR, complete recovery; GBS, Guillain-Barré syndrome; MFS, Miller Fisher syndrome; NCS, nerve conduction study; PGBS, paraparetic GBS; yr, years*.

### Search Results and Study Characteristics

**Figure 1:**
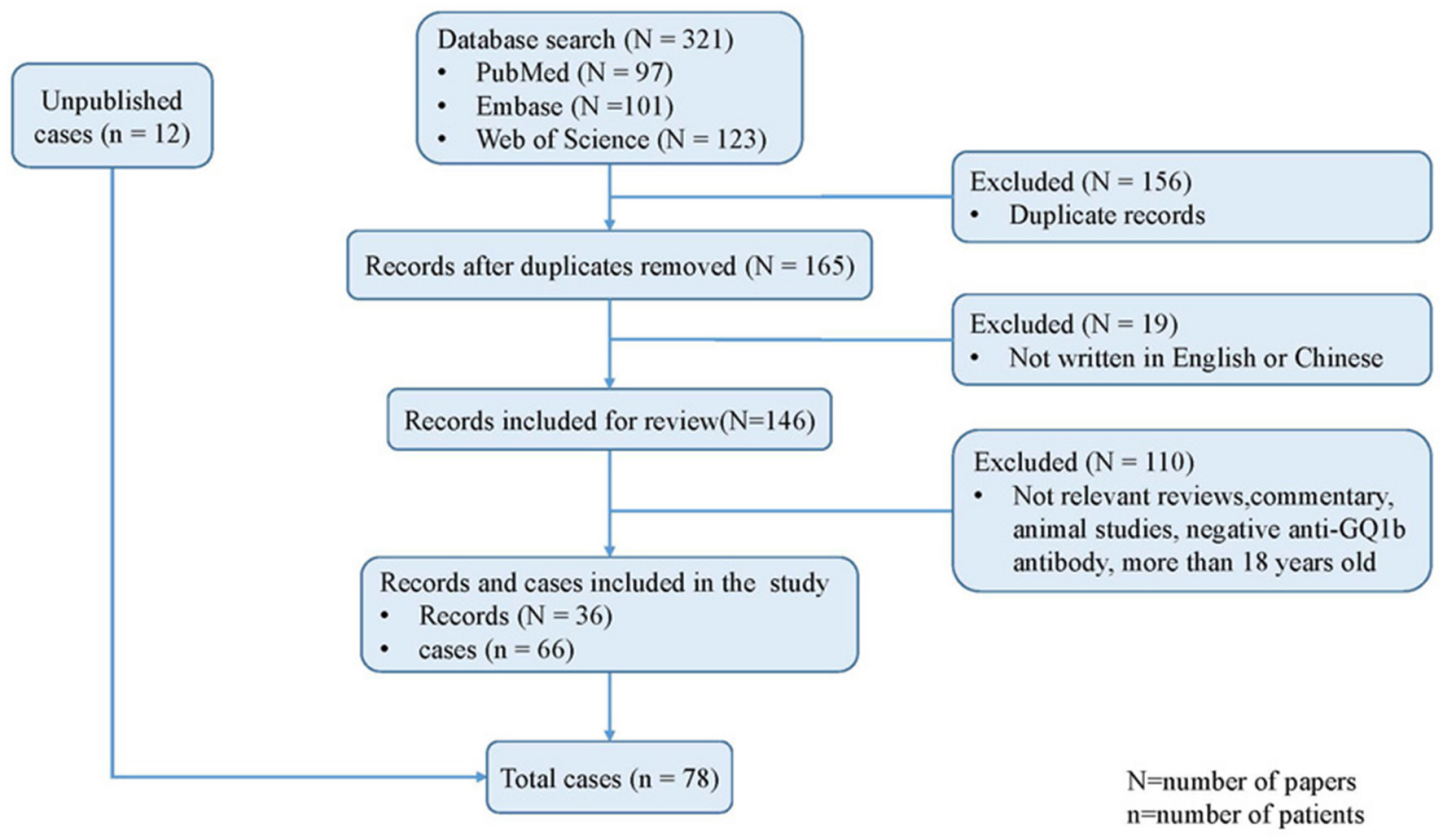
Algorithm of systematic review.

As shown in Figure 1, 321 citations in total were retrieved from the three databases. A combined method of manual and citation manager de-duplication was used to eliminate 156 duplicate citations and an additional 19 citations that were published in neither English nor Chinese, yielding a total of 146 citations for review. After abstract-level elimination and full manual review, a total of 36 papers reporting the detailed clinical data of 66 patients with anti-GQ1b antibody syndrome were identified and included. Supplementary Table 2 summarizes the detailed data of the published studies. All included studies were descriptive and retrospective. Thirty-three studies were case reports with <5 patients, and only one study presented >10 cases (10). However, the demographic data such as onset age and sex of the nine patients reported by Yoon et al. were incomplete (10), and the prognoses of another eight patients were absent (7, 11, 12). Therefore, some items had to be cited selectively.

Finally, a total of 78 patients were included in the present study, comprising the published studies and the unpublished data from two tertiary pediatric neurology centers in China.

### Demographic Characteristics

We analyzed the demographic characteristics of 69 patients. Due to the absence of associated data in the other nine patients, only two patients from the cohort of Yoon et al. (10) were included. In total, the onset age was 2–18 years old (median age, 10 years). The majority of patients (76.8%, 53/69) were aged >6 years. The patients were classified into four groups by age: toddler, 2–3 years (*n* = 4, 6%); preschool, 3–6 years (*n* = 12, 17%), school-age, 6–12 years (*n* = 27, 39%); adolescent, 12–19 years (*n* = 26, 38%). The male: female ratio was 48:21.

### Antecedent Infection

A total of 54 patients (69%) had antecedent infection or vaccination within 21 days prior to the onset of symptoms. Among them, upper respiratory tract infection (URTI) and acute gastroenteritis were the most common precedent illness, accounting for 35% (27 cases) and 24% (19 cases) of patients, respectively. Five patients (6%) were diagnosed separately with skin infection, ocular inflammation, chickenpox, herpes, or pneumonia. Two patient (3%) developed symptoms of fever. One patient (1%) had a history of vaccination.

### Phenotypic Spectrum

**Figure 2:**
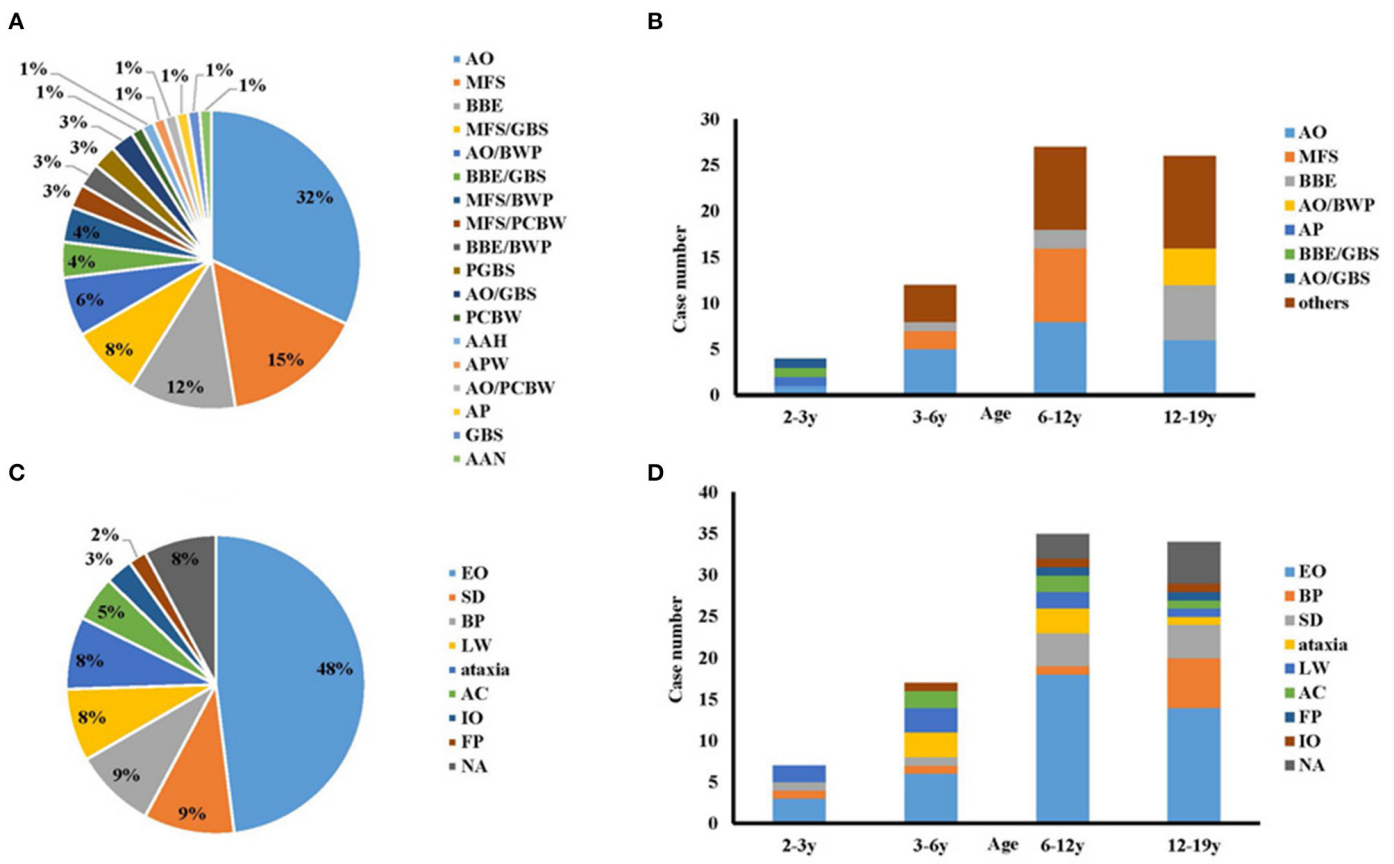
The onset symptoms and phenotypes of this cohort. **(A)** Phenotypes of anti-GQ1b antibody syndrome; **(B)** Subtypes categorized by age; **(C)** Onset symptoms of anti-GQ1b antibody syndrome in childhood; **(D)** Onset symptoms categorized by age. AAH, acute ataxic hypersomnolence; AAN, acute ataxic neuropathy; AC, altered consciousness; AO, acute ophthalmoparesis; AP, acute ptosis; APW, acute pharyngeal weakness; BBE, Bickerstaff brainstem encephalitis; BP, bulbar palsy; BWP, bifacial weakness with paraesthesias; EO, external ophthalmoplegia; FP, facial paralysis; GBS, Guillain-Barré syndrome; IO, internal ophthalmoplegia; LW, limb weakness; MFS, Miller Fisher syndrome; NA, not available; PCBW, pharyngeal-cervical-brachial weakness; PGBS, paraparetic GBS; SD, sensory disturbance.

The symptoms and signs of all 78 children included in the study were re-evaluated according to the new diagnosis classification (9). The distributions of the phenotypic diagnoses were (Figure 2A): AO, 25 cases (32%); MFS, 12 cases (15%); BBE, nine cases (12%); MFS-GBS overlap syndrome (MFS/GBS), six cases (8%); AO-bifacial weakness with paraesthesias (BWP) overlap syndrome (AO/BWP), five cases (6%); BBE-GBS overlap syndrome (BBE/GBS), three cases (4%); MFS-BWP overlap syndrome (MFS/BWP), three cases (4%); and others, 15 cases (19%). Fifty-four cases had only one phenotype, while 24 presented overlap syndromes.

Regarding the age profile of the phenotypic spectrum, BBE/GBS, acute ptosis (AP), AO, and AO-GBS overlap syndrome (AO/GBS) showed equal composition and accounted for one case each in toddlerhood (Figure 2B). By comparison, sole AO, MFS, and BBE were the most common phenotypes in the other three groups aged >3 years: preschool, 66.6% (*n* = 8); school-age, 66.6% (*n* = 18); adolescent, 46% (*n* = 12).

### Onset Symptoms

The most prevalent onset symptom was external ophthalmoplegia (EO), which affected 48% of the cases (Figure 2C). However, the distribution of the onset manifestations by age group differed (Figure 2D). In toddlers, the prevalence of EO was 43% (*n* = 3), followed by 29% (*n* = 2) with limb weakness (LW), 14% with bulbar palsy (BP, *n* = 1), and 14% with sensory disturbance (SD, *n* = 1). While in any groups aged > 3 years, EO represent the most common symptom. The second common one in the preschool, school-age, and adolescent groups was LW and ataxia (*n* = 6, 18%), SD (*n* = 4, 11%), and BP (*n* = 6, 18%), respectively.

### Anti-GQ1b Antibodies

Anti-GQ1b IgGs were serum-positive in 92% of the patients (72/78); another four cases (13, 14) consisting of two cases from our cohort (case 71 and 72, Supplementary Table 2) were anti-GQ1b IgM positive, and only two patients had no detailed Ig subtype (12, 15). Twenty-two cases were concurrently positive for other anti-ganglioside antibodies, and the most common was anti-GT1a antibody (55%, 12/22). Among these 12 cases, only three presented as BWP or PCBW with other subtypes. Up to 45% (10/22) of the patients with anti-IgGs beyond anti-GQ1b antibody had overlap syndrome: BBE/GBS (*n* = 2), AO/GBS (*n* = 2), MFS/GBS (*n* = 2), MFS-PCBW overlap syndrome (*n* = 1), MFS/BWP (*n* = 1), BBE-BWP overlap syndrome (*n* = 1), and AO/BWP (*n* = 1); another 12 patients had a sole phenotype: AO (*n* = 7), MFS (*n* = 3), Paraparetic GBS (*n* = 1), and GBS (*n* = 1). The earliest time to validation of the positive anti-GQ1b antibody was 1 day after onset.

### MRI, NCS, and CSF Analysis

Overall, MRI was performed for 68 patients. Among them, only 18% of cases (12/68) demonstrated abnormal signals on MRI: cerebral white matter, cerebellum, and brain stem lesions (*n* = 7; case 10, 20, 21, 34, 36, 37, 64; Supplementary Table 2) (10, 15–19), cranial nerve involvement (case 9, 35, 39; Supplementary Table 2) (10, 11, 20), myelitis-like demonstration (case 72; Supplementary Table 2), and enhanced meningeal signal (case 41; Supplementary Table 2) (21). Regarding the phenotype, five patients had either BBE alone or combined with other phenotypes, and three patients had MFS.

Forty-five patients underwent NCS; 60% (27 cases) had abnormal findings: reduced compound muscle action potential (13/27), reduced or disappeared sensory nerve action potential (11/27), prolonged latency (9/27), decreased conduction velocity (7/27), absent H reflexes (6/27), and reduced or disappeared F wave (4/24). Of these 27 patients, 14 were diagnosed with MFS, nine with GBS, and four with PCBW alone or combined with other phenotypes. Another three cases without peripheral nerve system involvement showed abnormal F waves and absent H reflex in two BBE cases (22) and one from our cohort (case 67; Supplementary Table 2), respectively, as well as decreased CV and abnormal F waves in one AP case (23).

A total of 87% of patients (68/78) underwent lumbar puncture (LP) and CSF studies. The LP time was 1–36 days after onset (median, 8 days). Twenty-three patients (34%) had elevated protein, defined as >45 mg/dL, and normal cell numbers, which indicated ACD. In the 23 cases, the time of ACD occurrence after symptom onset was 1–21 days (median, 8 days). The largest number of leukocytes and highest protein content in the CSF was 180 × 10^6^/L and 235 mg/dL, respectively (16).

**Figure 3:**
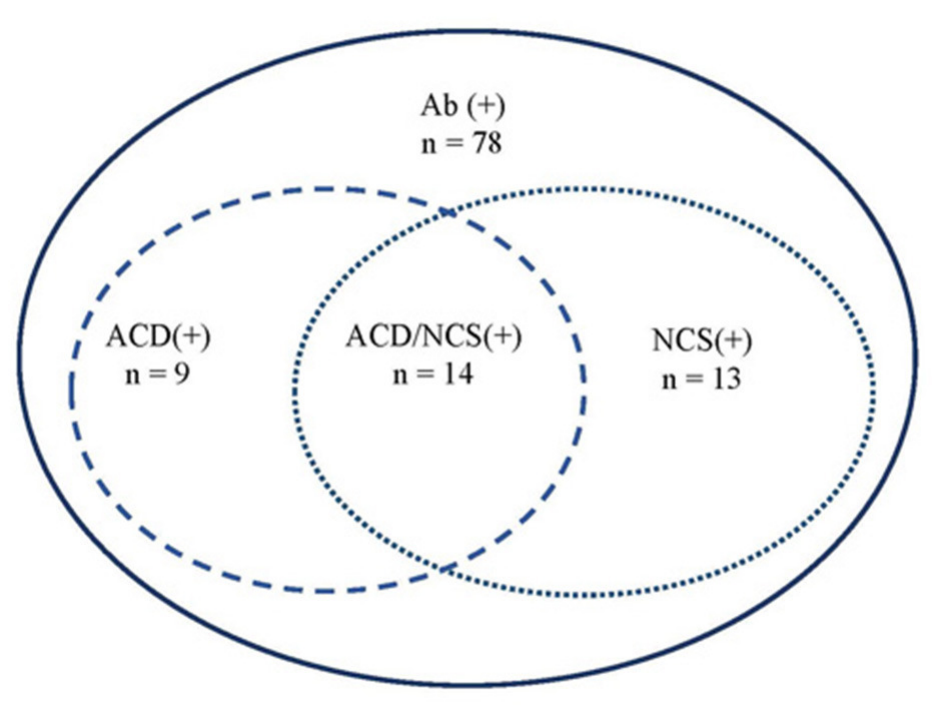
Correlation between ACD and NCS. Ab, anti-GQ1b antibodies; ACD, albuminocytologic dissociation; NCS, nerve conduction study.

Overall, 14 cases presented both abnormal NCS and ACD (Figure 3), accounting for 36% (14/39) of the patients who had undergone both LP and NCS.

### Treatment and Prognosis

During hospitalization, 42 patients were treated with IV immunoglobulin (IVIG) alone or combined with steroids or plasma exchange (PE). The other patients received PE, corticosteroids, or supportive therapy only. In the 70 cases with observational outcomes, 81% (57/70) recovered completely within the 1-year follow-up, and one even achieved full recovery at 7 days after the intervention (23). 56% (32/57) of the 57 cases were treated with IVIG alone or combined with steroids or PE, 33% (19/57) were treated with non-IVIG treatment. Another 13 cases showed incomplete recovery with follow-up spanning 20 days to 12 months since the treatment. Among them, 84% (*n* = 11) patients were aged >6 years. Likewise, AO (38%, 5/13) and MFS(23%, 3/13) were the most common phenotypes. 69% (*n* = 9) of cases had high titers of anti-GQ1b IgG antibody ranging from 1:500 to 1:12,800. Serum anti-GT1a IgG antibody and anti-GM1 IgG antibody were also, respectively, strong positive in one patient with MFS and the other with AO/GBS (case 58,59; Supplementary Table 2). 62% (8/13) patients were treated with IVIG alone or combined with other therapeutic interventions.

As shown in Table 3, Chi-square test and the fisher's exact test didn't find statistically significant between two groups in age, sex, preceding infection, phenotypes, CSF, NCS, cranial and spinal MRI, while the coefficient *p*-values for age and phenotype were statistically significant (*p* = 0.039, *p* = 0.191, respectively) by univariate screening. However, multifactorial binomial logistic regression showed that the two variables did not have significant predictive value for early improvement of up to 3 months.

**Table 3:** Comparison between complete and incomplete recovery patients.

	**CR(*n =* 27)**	**IR(*n =* 30)**	***p-*Value**
Age of onset			
2–3 yr	3/27 (11.1%)	1/30 (3.3%)	0.530
3–6 yr	6/27 (22.2%)	4/30 (13.3%)	0.595
6–12 yr	12/27 (44.4%)	11/30 (36.7%)	0.550
12–19 yr	6/27 (22.2%)	14/30 (46.7%)	0.054
Male:Female	17:10	23:7	0.259
Type of preceding infection			
URTI	12/27 (44.4%)	14/24^a^ (58.3%)	0.322
GI	8/27 (29.6%)	6/24^a^ (25.0%)	0.712
Others	3/27 (11.1%)	3/24^a^ (12.5%)	1.000
No infection	4/27 (14.8%)	1/24^a^ (4.2%)	0.421
Type of phenotypes			
AO	10/27 (37.0%)	6/30 (20.0%)	0.153
MFS	2/27 (7.4%)	5/30 (16.7%)	0.510
BBE	2/27 (7.4%)	7/30 (23.3%)	0.200
Other	13/27 (48.1%)	12/30 (40%)	0.536
ACD in CSF study	9/25^a^ (36.0%)	10/28^a^ (35.7%)	0.938
NCS abnormality	13/19^a^ (68.4%)	13/17^a^ (76.5%)	0.717
MRI abnormality	4/25^a^ (16.0%)	7/26^a^ (26.9%)	0.343
Treatment
IVIG only	11/27 (40.7%)	9/24^a^ (37.5%)	0.813
IVIG+ steriods	5/27 (18.5%)	5/24^a^ (20.8%)	1.000
Supportive care only	8/27 (29.6%)	6/24^a^ (25.0%)	0.712
Other treatments	3/27 (11.1%)	4/24^a^ (16.7%)	0.565

a *statistics using the number of patients who were available for each test; ACD, albuminocytologic dissociation; AO, acute ophthalmoplegia; BBE, Bickerstaff brainstem encephalitis; CR, complete recovery; CSF, cerebral spinal fluid; GI, gastroenteritis; IR, incomplete recovery; MFS, Miller Fisher syndrome; NCS, nerve conduction study; URTI, upper respiratory tract infection; yr, years*.

## Discussion

The main findings of the present study are summarized as follows: the most frequent pediatric phenotype of anti-GQ1b antibody syndrome is AO, rather than MFS in adulthood (3). Meanwhile, rarely reported overlapped syndromes, e.g., MFS/GBS, AO/BWP, and BBE/GBS, can also be seen in children. Additionally, the most common onset symptom in children is EO, similar to that in adulthood. The prevalence of ACD in the CSF (34%) was not as common as that in classic GBS. The majority of the patients have favorable prognosis, regardless of the onset age and phenotypic spectrum.

It has been well-acknowledged that antecedent infection of *Campylobacter jejuni* is the trigger factor for producing anti-GQ1b antibodies by molecular mimicry (2). In the present cohort, 35% of the patients had URTI prior to onset, while only 24% presented with acute gastroenteritis. In line with our findings, URTI (80%) was more common than gastroenteritis (14%) as prodromal infection in a cohort with 194 patients (3). Koga et al. also reported that *Haemophilus influenzae*-related URTI appeared to be more common than *C. jejuni* infection (21 vs. 14%) as antecedent infection in Miller Fisher syndrome (MFS) (24). The predominance of URTI over gastroenteritis supports the probable role of *H. influenzae* or other agents as triggering factors (9, 25).

The most frequent subtypes in the present cohort were sole AO (32%), followed by MFS (15%), BBE (12%), and MFS/GBS (8%), accounting for 67% of all cases. EO was the most common onset syndrome. By comparison, the most prevalent phenotype of anti-GQ1b antibody syndrome in adulthood is MFS (59%), followed by MFS/GBS (17%) and AO (10%) (26). The discrepancy of the onset presentation and phenotype may be ascribed to the lower content of GQ1b during early peripheral nerve development as well as its relatively higher enrichment in the paranodal regions of human oculomotor nerves as compared with the peripheral nerves (4, 27, 28).

With increasing awareness of the widely distribution of GQ1b antigen in the nerves system, accumulating phenotypes have been identified and novel syndromes are emerging. Visual deterioration, headache have been reported as initial symptomes (29, 30). In addition, acute vestibular syndrome (31), single ocular motor nerve palsy (32), pure bilateral Adie's pupils (33), and complete bilateral ophthalmoplegia with unilateral facial palsy (34) were also reported as new phenotypes of anti-GQ1b antibody syndrome. The comprehensive presentations of anti-GQ1b antibody syndrome are still to be expanded and summarized in both pediatric and adult patients.

GQ1b ganglioside is enriched in the paranodal regions of the extramedullary portion of the human oculomotor, trochlear, and Ia afferents in muscle spindles, and the abducens nerves, and probably in the ciliary ganglia (2, 28). It has also been detected in the cerebellar granular layer in rats (35). It has been speculated that anti-GQ1b antibody can access the brain stem parenchyma through the area postrema (36) and local disruption of the blood–nerve barrier near the roots of the cranial nerves (37). Molecular mimicry between GQ1b and lipo-oligosaccharides extracted from *C. jejuni* strains (38) and *H. influenzae* (39) has been demonstrated. All GBS subtypes were found in the cohort of children with anti-GQ1b antibody syndrome, which is indicative of the variable involvement of the central and peripheral nerve systems.

In the present cohort, 22 patients had other anti-ganglioside antibodies, the most common of which was anti-GT1a antibody (55%, 12/22). Anti-GT1a IgG cross-reacts with GQ1b in 75% of patients, and the clinical findings between sole serum-positive anti-GQ1b antibody and anti-GQ1b/GT1a antibody are similar (40). Our results also show that only 25% (*n* = 3) of patients presented cranial nerve and neck weakness as BWP and PCBW, which are the relative specific clinical manifestations of sole anti-GT1a IgG (40). In addition, only 45% (10/22) of the patients with anti-IgGs beyond anti-GQ1b showed overlap syndrome, which did not correspond to the distribution of positive antibodies. Notably, one patient (14) showed only AO, though the anti-GQ1b and anti-GM1 antibody were both positive. All the data indicated that the phenotypes of anti-GQ1b antibodies are heterogeneous and not always correlated specifically to the seropositive antibodies, and this may be associated with the differing complex-enhanced or complex-attenuated anti-GQ1b antibodies among patients and the different expression of gangliosides in different parts of the nervous system (26, 41).

CSF examination may be useful in cases of clinical uncertainty about diagnosis, especially to exclude other causes associated with CSF pleocytosis, such as infectious polyradiculitis, and acute poliomyelitis. ACD was found in only 34% of cases, which is much lower than 58% in adult patients (3) and 77% of pediatric GBS cases (42). Similarly, the percentage of abnormal NCS in the present cohort was less frequent than that in pediatric GBS cases [60 vs. 91% (42)] and in adult FS cases (74%) (43). Overall, 36% of cases (14/39) had both ACD in CSF and abnormal NCS. These data suggest that NCS may aid in improving the diagnosis of anti-GQ1b antibody syndromes without ACD in CSF, especially in the MFS-, GBS-, and PCBW-related spectrums.

Up to 11% of BBE cases and 1% of FS cases show abnormal signals on brain MRI (2). By comparison, MRI of 18% of cases in the present cohort showed lesions in the cerebellum, brainstem, cranial nerve, and spinal cord. Besides, white matter (10), basal ganglia (16), and periaqueductal area (18, 19) were also detected in the present cohort. Whether the lesions outside the enriched area of GQ1b expression are causative of anti-GQ1b itself requires further elucidation.

Randomized controlled trials on the anti-GQ1b spectrum overlapping with PCB or GBS have shown that IVIG and PE are efficacious for improving outcome (44). In the present study, therapy regimen was excluded by univariate screening for the factors related to short-term prognosis initially. Furthermore, we found neither of other factors including onset age, phenotypes, abnormailities of CSF and NCS was correlated significantly to the short-term prognosis. Despite the overall favorable prognosis of this entity, 13 cases showed incomplete recovery. And the majority were aged over 6 years, which may be associated with inadequate recovery based on the findings that the children with MFS showed more favorable prognosis than the adult (45). High titer of antibody has been reported to positively correlated with the severity of this disorder (46). Therefore, 69% of cases with higher titers of anti-GQ1b IgG antibody in this cohort possibly contribute to the poor outcome. However, the association of activities of the antibody with treatment responses and prognosis are still unclear thus far. As a result, the predictors of both short-term and long-term prognosis in pediatric anti-GQ1b antibody syndrome remain unclear. Given that the pathogenesis of anti-GQ1b antibody syndrome is similar to that of GBS, IVIG might remain the first-line treatment for this syndrome whether in adults or children from the aspect of short-term benefit (2).

The major limitation of our study is that all cases were from retrospective studies at different times, which biases the phenotype differentiation and prognosis because of the different observation courses and therapies. Besides, due to the rarity of the condition, the small sample makes it difficult to establish the differences among variant phenotypes in terms of precedent infection, auxiliary tests, and prognosis. The lack of anti-GQ1b IgG titers may also lead to the underestimate its' impact on the prognosis. Therefore, larger prospective multicenter studies are necessary for clarifying the clinical characteristics and evolution of anti-GQ1b antibody syndrome in children.

Here, we describe previously unreported 12 children with anti-GQ1b antibody syndrome, and summarize the clinical characteristics and evolution of a resultant 78 cases. Different from adult patients, AO and classic MFS are the most common phenotypes of anti-GQ1b antibody syndrome in childhood. The most prevalent onset symptom was EO. Immunotherapies including IVIG remain the most common treatments, and the majority of patients show good prognosis. The correlation of phenotype with other concomitant antibodies, onset age, laboratory tests, and prognosis remain to be explored further.

## Data Availability Statement

The original contributions presented in the study are included in the article/Supplementary Material, further inquiries can be directed to the corresponding author.

## Ethics Statement

The studies involving human participants were reviewed and approved by The Institutional Review Boards of Children's Hospital of Chongqing Medical of University and Shenzhen Children's Hospital. The patients/participants provided their written informed consent to participate in this study.

## Author Contributions

LCa: data collection and interpretation, follow-up of patients, and writing the manuscript. ZH: data collection, follow-up of patients, and writing the manuscript. JL: data collection, follow-up of patients, and manuscript revision. SH, LK, and LCh: data collection and follow-up of patients. YL: statistical analysis and data interpretation. TL: conception and design of the work, data interpretation, and manuscript revision. LJ: data interpretation and manuscript revision. All authors contributed to the article and approved the submitted version.

## Conflict of Interest

The authors declare that the research was conducted in the absence of any commercial or financial relationships that could be construed as a potential conflict of interest.
