# Supplementary material for: Clinical Characterization of Anti-GQ1b Antibody Syndrome in Childhood
Source: Front Pediatr. 2021 Apr 29;9:649053. doi: 10.3389/fped.2021.649053 (PMC8116501; doi:10.3389/fped.2021.649053)
Supplement: Supplementary file 1 [file Table_1.DOCX]

| Table-e 1 Clinical and laboratory findings in the Chinese cohort | | | | | | | | | | | | |
| --- | --- | --- | --- | --- | --- | --- | --- | --- | --- | --- | --- | --- |
| Case no. | 67 | 68 | 69 | 70 | 71 | 72 | 73 | 74 | 75 | 76 | 77 | 78 |
| Sex/age | F/13y | M/6y4m | F/6y5m | M/12y5m | F/2y10m | F/3y3m | M/4y | M/2y6m | M/9y1m | M/7y3m | M/8y6m | M/3y4m |
| Antecedent infection | URI | URI | URI | - | URI | - | - | URI | URI | - | - | URI |
| Latency | 7d | 30d | 7d | - | 2d | - | - | 15d | 2d | - | - | 7d |
| Initial symptom | EO | LW, SD | SD | EO, BP | LW, SD | LW, BP | Ataxia, SD | EO, LW, BP | EO | LW | EO | EO, Ataxia |
| Phenotype | BBE | PGBS | GBS | MFS/BWP | BBE/GBS | BBE/GBS | MFS | AO/GBS | MFS/PCBW | PGBS | MFS | MFS |
| Tendon reflex | Absent | Normal | Absent | Absent | Absent | Absent | Absent | Hyperreflexia | Absent | Absent | Absent | Absent |
| Days to nadir | 2d | 1d | 7d | 10d | 2d | 1d | 5d | 1d | 3d | 2d | 2d | 3d |
| Antibodies  (days after onset) | Anti-GQ1b IgG/21d | Anti-GQ1b/  GD1b/GT1b/GM1/GM2 IgG/17d | Anti-GQ1b/ GT1a/GD3 IgG/8d | Anti-GQ1b IgG/9d | Anti-GQ1b/  GD1b/GM1/GM2 IgM; Anti-GM2 IgG/11d | Anti-GQ1b/ GD1b/GT1b /GM1/GM2 IgM/8d | Anti-GQ1b IgG /16d | Anti-GQ1b/  Sulfatide/GT1a/GM1 IgG; Anti-Sulfatide/ GT1a/GM1 IgM /3d | Anti-GQ1b/ GT1a IgG/7d | Anti-GQ1b IgG/21d | Anti-GQ1b/GT1a IgG; Anti-GM1/  GM3 IgM/8d | Anti-GQ1b IgG; Anti-GM1 IgM/6d |
| CSF analysis  (days after onset) | 10d | 16d | 7d | 9d | 2d | 3d | 6d | 2d | 9d | 21d | 8d | 10d |
| Cell count (*10^6^/L) | 10 | 20 | 0 | 0 | 5 | 2 | 1 | 1 | 0 | 0 | 1 | 10 |
| Protein (g/L) | 0.41 | 0.54 | 0.35 | 0.86 | 0.58 | 0.91 | 0.76 | 0.27 | 0.28 | 1.1 | 0.23 | 1.17 |
| NCS | PL of F wave;  H reflex (-) | RA of CMAP; F wave(-);SNAP (-) | H reflex (-) | PL of F wave and H reflex; SNAP (-) | RF of CMAP;  RA of SNAP; slowed SCV; F wave (-) | PL and RA of CMAP; slowed MCV; SNAP (-); RF of F wave | PL and RA of CMAP; slowed MCV; SNAP (-) | RA of CMAP | H reflex (-) | PL of CMAP | Slowed SCV | H reflex (-) |
| Cranial MRI | Unremarkable | Unremarkable | Unremarkable | Slightly enlarged left lateral ventricle | Unremarkable | Punctate abnormal signal in the anterior horn of the left lateral ventricle | Unremarkable | Unremarkable | Unremarkable | Unremarkable | punctate abnormal signal in the left frontal lobe | Unremarkable |
| Spinal cord MRI | unmremarkable | epidural effusion at chest 2-9 vertebral level | Unremarkable | Unremarkable | NA | myelitis-like demonstration on thoracic spinal cord. | NA | Unremarkable | Unremarkable | Unremarkable | NA | NA |
| Treatment | IVIG+DEX→PRD | IVIG | IVIG | IVIG+MP→,PRD | IVIG, MP | IVIG | IVIG→PRD | IVIG, MP | IVIG+MP→,PRD | IVIG | IVIG | IVIG+MP→,PRD |
| Prognosis | CR within 90d | CR within 30d | CR within 15d | CR within 90d | CR within 32d | CR within 51d | CR within 17d | CR within 210d | CR within 60d | CR within 180d | CR within 90d | CR within 180d |
| Abbreviations: AO: acute ophthalmoplegia; BBE: Bickerstaff brainstem encephalitis; BP: Bulbar palsy; BWP: Bifacial weakness with paranesthesia; CMAP: compound muscle action potential amplitude; CR: complete recovery; D: day DEX: dexamethasone: external ophthalmoplegia ;F: female; GBS: Guillain–Barré syndrome; IVIG: Intravenous immunoglobulin; LW: Limb weakness; M: male; MCV: motor nerve conduction velocity; MFS: Miller Fisher syndrome; MP: methylprednisolone ;NA: not available; PGBS: paraparetic GBS; PL: Prolonged latency; PRD: [prednisone](https://cn.bing.com/dict/search?q=prednisone&FORM=BDVSP6&mkt=zh-cn);RA:reduced amplitude; RF: reduce frequency; SCV: sensory nerve conduction velocity; SD: : Sensory disturbance; SNAP: sensory nerve action potentials; SV: slowed velocity; URI: Upper respiratory infection. | | | | | | | | | | | | |
